# Annual acknowledgement of manuscript reviewers

**DOI:** 10.1186/s13104-016-1958-x

**Published:** 2016-03-14

**Authors:** Christopher Foote

**Affiliations:** BioMed Central, Floor 6, 236 Gray’s Inn Road, London, WC1X 8HB UK

The editors of *BMC Research Notes* would like to thank all our reviewers who have contributed to the journal in Volume 8 (2015).

**Salman Abbasifard**

USA

**Ahmed Abdel-Hadi**

Egypt

**Nourtan Abdeltawab**

Egypt

**Hamza Abdulghani**

Saudi Arabia

**Khatijah Binti Abdullah**

Malaysia

**Hala Abdullahi**

Sudan

**Hiroyuki Abe**

Japan

**Kohtaro Abe**

Japan

**Wonder Abotsi**

Ghana

**Carla Abouzahr**

Australia

**Amit Acharya**

USA

**Sonu Acharya**

India

**Cristina Acin**

Spain

**Jessica Ackert**

USA

**Ishag Adam**

Sudan

**Lisa Adams**

USA

**Olusegun Adebami**

Nigeria

**Clement Adebamowo**

USA

**Adeolu Adedapo**

Nigeria

**Davies Adeloye**

UK

**Jeremy Adler**

USA

**Joe Aduse-Opoku**

UK

**Mukesh Agarwal**

United Arab Emirates

**Suresh K. Agarwal Jr.**

USA

**Manjeet Aggarwal**

India

**Riaz Agha**

UK

**Stamatis Agiovlasitis**

USA

**Chimere Agomo**

Nigeria

**Bernardo Agostini**

Brazil

**Vinita Agrawal**

India

**Kees Ahaus**

The Netherlands

**Mohamed Azmi Ahmad Hassali**

Malaysia

**Md Firoz Ahmed**

UK

**Saifuddin Ahmed**

USA

**Wisal Ahmed**

Sudan

**Muhammad Yakoob Ahmedani**

USA

**Myung-Ju Ahn**

South Korea

**Riitta Ahonen**

Finland

**Jessie Ahroni**

USA

**Tom Ainscough**

UK

**Elizabeth Aitken**

Australia

**Atem Ajong**

Cameroon

**Nadia Akawi**

UK

**Tetsu Akimoto**

Japan

**Adewale Akinsola**

Nigeria

**Olufunke Alaba**

South Africa

**Olufemi Alabi**

USA

**Souza Alain**

Gabon

**Hasanat Alamgir**

USA

**Sinaa Al-Aqeel**

Saudi Arabia

**Sandra Alba**

The Netherlands

**Costantine Albany**

USA

**Jennifer S. Albrecht**

USA

**Tara Albrecht**

USA

**Sol Aldrete**

USA

**Sami Al-Dubai**

Malaysia

**Paul Alele**

Uganda

**Abebe Alemu**

Ethiopia

**David Alexander**

Canada

**Camille Alexis-Garsee**

UK

**Ana Alfirevic**

UK

**Murad Al-Holy**

Jordan

**Mohammad Ali**

USA

**Shahzad Ali**

Pakistan

**Bachti Alisjahbana**

Indonesia

**Mohamed Al-Khaled**

Germany

**Ahmed Alkhateeb**

USA

**Jacqui Allen**

New Zealand

**Norrina Allen**

USA

**Stephanie Alley**

Australia

**Anna Birna Almarsdóttir**

Denmark

**Hassan Almoazen**

USA

**Gad Alon**

USA

**Fahad Alosaimi**

Saudi Arabia

**Hamzah Alqadiri**

Jordan

**Khalid Al-Rubeaan**

Saudi Arabia

**Ahmed Alsuwaidi**

United Arab Emirates

**Teatske Altenburg**

The Netherlands

**Illimar Altosaar**

Canada

**Ruben Alvero**

USA

**Joana Alves**

Portugal

**Sharif Aly**

USA

**Tarek Amin**

Egypt

**Soheila Aminimoghaddam**

Iran

**Riccardo Amorati**

Italy

**Jaime Amorim Santos**

Brazil

**Emmanuelle Amoros**

France

**Raghupathy Anchala**

India

**Jessica Ancker**

USA

**Aase Bengaard Andersen**

Denmark

**Erik Andersen**

USA

**Stig Andersen**

Denmark

**Alex Anderson**

USA

**Alisha Anderson**

Australia

**Adolfo Andrade-Cetto**

Mexico

**Genevieve Andre-Fontaine**

France

**Solange Andreoni**

Brazil

**Steven Andrews**

USA

**Banu Anlar**

Turkey

**Daniel Antonius**

USA

**Binu Antony**

Saudi Arabia

**James Aquavella**

USA

**Andreza Maria Aranha**

Brazil

**Vasanthy Arasaratnam**

Sri Lanka

**Mohammad Arbabi**

Iran

**Thiago Ardenghi**

Brazil

**Jesse Ariasamy**

Malaysia

**Mohammad Arif**

USA

**Anne Arikian**

USA

**Ahmet Arman**

Turkey

**Miguel Armengot**

Spain

**John A. L. Armour**

UK

**Sophie Arnaud-Haond**

France

**Filip Arnberg**

Sweden

**Benjamin Arnold**

USA

**Forest Arnold**

USA

**Oscar Arrieta**

Mexico

**Abdullah Arslan**

USA

**Shervin Assari**

USA

**Shervin Assassi**

USA

**Mitra Assemi**

USA

**Ricardo Ataíde**

Portugal

**Matias Attene Ramos**

USA

**Christopher Auger**

Canada

**Ben Augustine**

USA

**Nicanor Austriaco**

USA

**Sayyed Ayatollahi**

Iran

**Vasco Azevedo**

Brazil

**Akira Babazono**

Japan

**Lutz Bachmann**

Norway

**Floor Backes**

USA

**Ahmed Badar**

Saudi Arabia

**Farid Badria**

Egypt

**Taeok Bae**

USA

**Prasanta K. Bag**

India

**Haibo Bai**

USA

**Lubna Baig**

Pakistan

**Henry Bailey**

Trinidad and Tobago

**Michael Bailey**

USA

**Patricia Bailey**

USA

**Leonardo Baiocchi**

USA

**Amy Baird**

USA

**Indira Bairy**

India

**Meenakshi Bajpai**

India

**Martin Bak**

Denmark

**Judith Baker**

USA

**Shankar Bakkannavar**

India

**Tamas Bakonyi**

Austria

**Giorgos Bakoyannis**

USA

**Ashraf Bakr**

Egypt

**Chinthaka Balasooriya**

Australia

**Madhan Balasubramanian**

Australia

**Udeni Balasuriya**

USA

**Marzia Baldereschi**

Italy

**Jennifer Balkus**

USA

**Pwaveno Bamaiyi**

Malaysia

**Niaz Banaei**

USA

**Eliane Bandinelli**

Brazil

**Samprit Banerjee**

USA

**Didi Bang**

Denmark

**Carsten Bantel**

Germany

**Luigi Barberini**

Italy

**Perry Barboza**

USA

**Nêmora Barcellos**

Brazil

**Suzanne Barker-Collo**

New Zealand

**Patrick Barlow**

USA

**Andrew Barnes**

Australia

**Benjamin Bar-Oz**

Israel

**Cristina Barrero Sicilia**

UK

**Bruce Barrett**

USA

**Trayambak Basak**

USA

**Tom Bashford**

UK

**Amal Bashir**

Sudan

**Shahindokht Bassiri Jahromi**

Iran

**Bristi Basu**

UK

**Veronique Bataille**

UK

**Laia Batalla-Carrera**

Spain

**Giacomo Batignani**

Italy

**Bruno Batista**

Brazil

**Maximilian Baumann**

Germany

**Angela Bazzi**

USA

**Artur Beberok**

Poland

**Nicolas Bech**

France

**Jeffrey Beck**

USA

**Branka Bedenic**

Croatia

**Giorgio Bedogni**

Italy

**Prakash Behere**

India

**Vicente Jr. Belizario**

Philippines

**Stefanos Bellos**

Greece

**Yeshambel Belyhun**

Ethiopia

**Marije Benedictus**

The Netherlands

**Raja Benkirane**

Morocco

**Brian Bennett**

USA

**Philip Benson**

UK

**Fred Bercovitch**

Japan

**Katherine Berg**

Canada

**Peter Bergman**

Sweden

**César Bergoli**

Brazil

**James Berkley**

Kenya

**Kathryn Bernard**

Canada

**Eric Bershad**

USA

**Marian Betz**

USA

**Teresa Beyen**

Ethiopia

**S. Bhat**

India

**Jatinder Bhatia**

USA

**Deepak Bhatnagar**

India

**Dhrubajyoti Bhattacharya**

USA

**Shoumo Bhattacharya**

UK

**Rajendra Bhimma**

South Africa

**Elizabeth Bhoj**

USA

**Mahmood Bhutta**

UK

**Godfrey Biemba**

Zambia

**Ghulam Bilal**

Canada

**Jalal Bilal**

Saudi Arabia

**Saleh Binsaleh**

Saudi Arabia

**Samiran Bisai**

India

**Subrata Biswas**

Bangladesh

**Marcio Sommer Bittencourt**

USA

**Peyman Björklund**

Sweden

**Kirsten Black**

Australia

**Nancy Black**

Canada

**David Blackbourn**

UK

**Polina Blagojevic**

Serbia

**Holly Blake**

UK

**Amy Blakemore**

UK

**Lucas Blanton**

USA

**Joaquin Blaya**

USA

**Jeanet Blom**

The Netherlands

**Andrew Blum**

USA

**Vincent Boama**

Qatar

**Franck Boccara**

France

**Dankmar Boehning**

UK

**J. Ties Boerma**

Switzerland

**Jonathan Bogan**

USA

**Chrysi Bogiatzi**

Canada

**Jonathan Bohbot**

Israel

**Ioannis Boletis**

Greece

**Aline Boligon**

Brazil

**Alexander Bolshoy**

Israel

**Stephan Bongard**

Germany

**Ann Bonner**

Australia

**Sebastiano Bonventre**

Italy

**Matthias Bopp**

Switzerland

**Victor Borja-Aburto**

Mexico

**Stefan Bösner**

Germany

**Anne-Marie Bostrom**

Sweden

**Rami Bou Khalil**

France

**Mohamed Bouattour**

France

**Ons Bouchami**

Portugal

**Stefanos Boukovalas**

USA

**Matthew Boulton**

USA

**Mohamed Boutaayamou**

Belgium

**Jean-Marie Bouteiller**

USA

**Jon P. Boyle**

USA

**James Bradeen**

USA

**Leslie Bradford**

USA

**Michael Bradley**

Canada

**Ylisabyth Bradshaw**

USA

**William Brady**

USA

**Mirko Brandes**

Germany

**Andy Brass**

UK

**Elizabeth Braunlin**

USA

**Mary-Lynn Brecht**

USA

**Anna-Kristin Brettschneider**

Germany

**Michael Brewer**

USA

**William Brieger**

USA

**Johanna Brinkel**

Ghana

**Dustin Brisson**

USA

**Maurizio Francesco Brivio**

Italy

**Michael Bronze**

USA

**Marie Brossard-Racine**

Canada

**Carlos Brotons**

Spain

**Chris Brown**

New Zealand

**Joe Brown**

USA

**Norbert Bruggemann**

Denmark

**Silvio Brugger**

USA

**Kevin Brumund**

USA

**Tim Brusseau**

USA

**Dag Bruusgaard**

Norway

**Joseph Bryant**

USA

**Maria Bryant**

UK

**William Bryson**

USA

**Yiwen Bu**

USA

**Tomas Buchler**

Czech Republic

**Hikmet Budak**

Turkey

**Stephane Buhler**

Switzerland

**Nicola Burgess**

UK

**Monica Burnett**

USA

**Richard Burns**

Australia

**Joan Busfield**

UK

**Suk Ho Byeon**

South Korea

**Joshua Byrnes**

Australia

**Maria Cabanillas**

USA

**Alberto Caban-Martinez**

USA

**Florence Cabot**

USA

**Giuseppina Caggiano**

Italy

**Carlo Cagini**

Italy

**Chunhui Cai**

USA

**Adriana Calderaro**

Italy

**Michael S. Calderwood**

USA

**Tudor Calinici**

Romania

**Sharon Cameron**

UK

**Ruth Campbell**

USA

**Ahmet Selçuk Can**

Turkey

**Pietro Canetta**

USA

**Huansheng Cao**

USA

**Kai Cao**

USA

**Maurizia Capuzzo**

Italy

**Angela Carberry**

Australia

**Nikola Carboni**

Italy

**Sharon Card**

Canada

**Misericordia Carles**

Spain

**JanCarline**

USA

**Lucas Carr**

USA

**Joao Andre Carrico**

Portugal

**Andres Carrillo**

USA

**Michael Carter**

USA

**Petros Carvounis**

USA

**Sandra Cascio**

USA

**Alejandra Castanon**

UK

**Ricardo Castillo-Neyra**

Peru

**Maria Rita Castrucci**

Italy

**Jean-Baptiste Cazier**

UK

**Colleen Cebulla**

USA

**Peter Cegielski**

USA

**Marco Cei**

Italy

**Filipe Ceia**

Portugal

**Nuno Cerca**

Portugal

**Alexandru Cernat**

UK

**Martin Cernvall**

UK

**Sureka Chackrewarthy**

Sri Lanka

**Sarabjit Chadha**

India

**Benjamin Chaffee**

USA

**Lay Ching Chai**

Malaysia

**Anindita Chakrabarty**

India

**Soumitesh Chakravorty**

USA

**Mary Chambers**

UK

**Alexandre Chan**

Singapore

**Heng Choon Chan**

Hong Kong

**Martin C. W. Chan**

China

**Ananda Chandrasekara**

Australia

**Theresa Chang**

USA

**Alan Channing**

South Africa

**Brad Chapman**

USA

**Keyne Charlot**

France

**Narattaphol Charoenphandhu**

Thailand

**Lisa Chasan-Taber**

USA

**Aparna Chaudhari**

India

**Luis Chávez-Sánchez**

Mexico

**James Chen**

USA

**Jin Chen**

USA

**Yabin Chen**

USA

**Yen-Hsu Chen**

Taiwan

**Benoît Chénais**

France

**Jin-Quan Cheng**

China

**Keding Cheng**

Canada

**Thomas Chertemps**

France

**Teris Cheung**

Hong Kong

**Gary Chew**

Australia

**Munashe Chigerwe**

USA

**Jun Chih**

Australia

**Weng Yee Chin**

Hong Kong

**Siewmooi Ching**

Malaysia

**Vitoon Chinswangwatanakul**

Thailand

**Shubhada Chiplunkar**

India

**Jennifer Chipps**

South Africa

**Parkson Lee-Gau Chong**

USA

**Shuvasish Choudhury**

India

**Shubhajit Chowdhury**

India

**Mette Christiansen**

Denmark

**Peer Christiansen**

Denmark

**Nicola Christie**

UK

**Kyproula Christodoulou**

Cyprus

**Yi-Ping Chuang**

Taiwan

**Ampaiwan Chuansumrit**

Thailand

**Anita Chudecka-Glaz**

Poland

**Benjamin Chung**

USA

**Antonella Cianferoni**

USA

**Arrigo Cicero**

Italy

**Mehmet Ulas Cinar**

Turkey

**Christian Clarenbach**

Swaziland

**Bart Clarke**

USA

**Rui Claro**

Portugal

**Jill Clendon**

New Zealand

**Rosemary Clerehan**

Australia

**Jackie Cliff**

UK

**Lisa Clifford**

USA

**Lei Clifton**

UK

**Rachel Cloutier**

USA

**John Codrington**

Australia

**Tracey Coffey**

UK

**Alwyn Cohall**

USA

**Adam Cohen**

USA

**Bernard Cohen**

USA

**Bevin Cohen**

USA

**Steven A. Cohen**

USA

**William Coleman**

USA

**Yvonne Colgrove**

USA

**Charlotte Colvin**

USA

**Pierre Comizzoli**

USA

**Kim Connelly**

Canada

**Barbara Conner-Spady**

Canada

**Werner Conradie**

South Africa

**Cris Constantinescu**

UK

**Despina Contopoulos-Ioannidis**

USA

**Jane Conway**

Australia

**Mary Coolbaugh-Murphy**

USA

**Geoffrey Cooper**

USA

**Kerry Cooper**

USA

**Robin Cooper**

USA

**Lawson Copley**

USA

**Brent Copp**

New Zealand

**Cynthia Corbett**

USA

**Patricia M. Corby**

USA

**Marisa Cordella**

Australia

**Bert Cornelius**

The Netherlands

**Kerstin Cornils**

Germany

**Stephen Cornish**

Canada

**Ricardo Correa**

USA

**Germina Cosma**

Romania

**Luciane Costa**

Brazil

**Elizabeth Cotter**

USA

**James Couch**

USA

**Helene Cousin**

USA

**Diego Coutinho**

Brazil

**Michael Cox**

UK

**Thomas Cox**

Denmark

**Chiquito Crasto**

USA

**Gregory Crawford**

Australia

**Lynsey Cree**

New Zealand

**Deidra Crews**

USA

**Alexis Criscuolo**

France

**Lory Saveria Crocè**

Italy

**Andrew Croftys**

Japan

**Alan Cross**

USA

**Paul Cross**

UK

**Margaret Cruickshank**

UK

**Kimberley Cullen**

Canada

**Joana Cunha-Cruz**

USA

**Maria Paula Curado**

France

**Nicole Dabbs**

USA

**Akram Da’Darah**

USA

**Dave Dagnan**

USA

**Wendy Dahl**

USA

**Michael Daily**

USA

**Katie Dainty**

Canada

**Gavin Daker-White**

UK

**Jacob Dal-Bianco**

USA

**Ruben Dammers**

The Netherlands

**Fortunato D’Ancona**

Italy

**Cem Dane**

Turkey

**Rachel Dankner**

Israel

**Georges Daoud**

Lebanon

**Kristin Darin**

USA

**Azmi M. G. Darwazeh**

Jordan

**Krishanu Das**

India

**Susmita Datta**

USA

**Giuseppe Dattilo**

Italy

**Seema Daud**

Pakistan

**Robertson Davenport**

USA

**Michael David**

USA

**Alec Davidson**

USA

**Cally Davies**

Canada

**Eric Davies**

USA

**William Davies**

UK

**Ian Davis**

Australia

**Bratati De**

India

**Helen De Cieri**

Australia

**Costantino De Giacomo**

Italy

**Natasja M. S. de Groot**

The Netherlands

**Daisy D. De Leon**

USA

**Cristiano De Marchis**

Italy

**John Manuel de Oliveira e Silva Rodrigue**

Portugal

**Emilio Debess**

USA

**Sharon Deem**

USA

**Abraham Degarege**

Ethiopia

**Howard Degenholtz**

USA

**Louis Dehner**

USA

**Olivier Dejardin**

France

**Clare Delany**

Australia

**Anna Delprato**

France

**Andrea DeLuca**

USA

**Stephanie DeLuca**

USA

**Stefaan Demarest**

Belgium

**Maria Demetriou**

Greece

**Meaza Demissie**

Ethiopia

**Craig Denegar**

USA

**Swapnil Jayant Dengale**

India

**Ljiljana Denic**

Serbia

**Sarang Deo**

India

**Sarah Derrett**

New Zealand

**Yadeta Dessie**

Ethiopia

**Niveditha Devasenapathy**

India

**Mathias Devreese**

Belgium

**Anil Kumar Dhull**

India

**Anna Maria Di Giacomo**

Italy

**Albert Diefenbacher**

Germany

**Brenda Diergaarde**

USA

**Brian Diffey**

UK

**David Dimmock**

USA

**Michaela Dinan**

USA

**Bulent Dinc**

Turkey

**Jie Ding**

USA

**Wei-Qun Ding**

USA

**Xinqiang Ding**

USA

**Gillian Dite**

Australia

**Aviv Dombrovsky**

Israel

**Ferenc Domoki**

Hungary

**Xiao-Qiao Dong**

China

**Ravi Kiran Donthu**

USA

**Evan Doran**

Australia

**Anne Dorrance**

USA

**David Douches**

USA

**David Dowdy**

USA

**Kristian Doyle**

USA

**Cinthia Drachenberg**

USA

**Michel Drancourt**

France

**Cornelia Drees**

USA

**Jonathan Drennan**

UK

**Clemens Drenowatz**

USA

**Valderi Dressler**

Brazil

**Lubos Drgona**

Slovakia

**Raquel Duarte**

Portugal

**Whasley Duarte**

Brazil

**Andres Duarte-Rojo**

USA

**Pradeep Dudeja**

USA

**Edward Dudley**

USA

**Akilah Dulin Keita**

USA

**Denis J. Dupré**

Canada

**Christine Durand**

USA

**Ricardo Durlach**

Argentina

**Zachary Dwight**

USA

**Padmanabh Dwivedi**

India

**Michael Dwyer**

USA

**David Dyer**

USA

**Mathew Eapen**

Australia

**Jaya Earnest**

Australia

**Deren Eaton**

USA

**Richard Edmondson**

UK

**Rosato Edoardo**

Italy

**Dylan Edwards**

UK

**Paul Edwards**

USA

**David Eisenman**

USA

**Husamettin Ekici**

Turkey

**Abel Ekiri**

USA

**Alison El Ayadi**

USA

**John Elefteriades**

USA

**Targ Elgzyri**

Sweden

**Liselotte Schäfer Elinder**

Sweden

**Kate Ellacott**

UK

**Amanda Elliott**

USA

**Rosalind Elliott**

Australia

**Louisa Ells**

UK

**David Ellwood**

Australia

**Josep Ma Elorza-Ricart**

Spain

**Maged El-Setouhy**

Saudi Arabia

**Hany Elsheikha**

UK

**Ifeoma Emodi**

Nigeria

**Charis Eng**

USA

**Yvonne Engels**

The Netherlands

**James C. Engert**

Finland

**Anthony Enimil**

Ghana

**Elena Y. Enioutina**

USA

**Serpil Erdogan**

Turkey

**Kacey Ernst**

USA

**Heather Erwin**

USA

**Julio César Escalona Arranz**

Cuba

**Cam Escoffery**

USA

**Laila Espindola**

Brazil

**Musthafa Mohamed Essa**

Oman

**Johan Esterhuizen**

UK

**Enyi Etiaba**

Nigeria

**Michael Evangeli**

UK

**Alison Evans**

USA

**Charlotte Evans**

UK

**Denise Evans**

South Africa

**Jennifer Evans**

UK

**Charles Ezenduka**

Nigeria

**Bonto Faburay**

USA

**Pamela S. Fahs**

USA

**Carolyn Fang**

USA

**Qiyin Fang**

Canada

**Brian Faragher**

UK

**Khaleel Fareed**

UK

**Laura Fariselli**

Italy

**Evangelia Farmaki**

Greece

**A. Darise Farris**

USA

**Melissa Fath**

USA

**E. Vincent Faustino**

USA

**Frank Federico**

USA

**Brunhilde Felding-Habermann**

USA

**Marcia Feldkamp**

USA

**Steven Feldman**

USA

**Berhanu Feleke**

Ethiopia

**Daolun Feng**

China

**Gaoqian Feng**

Australia

**Hao Feng**

USA

**Liang Feng**

USA

**Quan Zhou Feng**

China

**Lia Fernald**

USA

**Maria Fernandez Tome**

Argentina

**Abdullah Feroze**

USA

**Giovanna Ferraioli**

Italy

**Pedro Ferreira**

Portugal

**Stefanie Ferreri**

USA

**Fernando Ferrero**

Argentina

**Marie Ferrua**

France

**Eric Fevre**

UK

**David Field**

UK

**Gregg Fields**

USA

**Albert Figueras**

Spain

**Helen Fillmore**

UK

**Suzanne Filteau**

UK

**Yavuz Fındık**

Turkey

**Ellen Fineout-Overholt**

USA

**Jeffrey Finnie**

South Africa

**Giulia Fiscon**

Italy

**Elisabeth Fitzek**

USA

**Gerry Fitzgerald**

Australia

**Colin Fitzsimmons**

UK

**Shaun Flint**

UK

**Sonia Flores**

USA

**Stephen Fong**

USA

**Marianna Fontana**

UK

**Frode Forland**

Norway

**Cynthia Formosa**

Malta

**Alice Forster**

UK

**Robert Foss**

USA

**Margaret Foster**

USA

**Bernard Fourie**

South Africa

**David Fournier**

France

**Denis Fournier**

Belgium

**Aaron Fox**

USA

**Bart Fraaije**

UK

**Ângela França**

Portugal

**Richard Francis**

Australia

**Jose R. Franco**

Switzerland

**Fritz Francois**

USA

**Scott Franklin**

USA

**Filippo Fratini**

Italy

**John Frean**

South Africa

**Lynetta Freeman**

USA

**Michael Freeman**

USA

**Carolina Freire**

Brazil

**Helen French**

Ireland

**Martyn French**

Australia

**Kevin Friedman**

USA

**Patrizia Froesch**

Switzerland

**Bastian Fromm**

Norway

**Caren Frost**

USA

**John Fruehauf**

USA

**Philip Frykman**

USA

**Manuel Fuentes**

Spain

**Ezequiel Fuentes-Panana**

Mexico

**Alexander Fuhrmann**

USA

**Kevin Fuji**

USA

**Hiroyuki Fujiwara**

Japan

**Tomoko Fukada**

Japan

**Hirokata Fukushima**

Japan

**Franco Fulciniti**

Switzerland

**Jennifer Furin**

USA

**John Furler**

Australia

**Daniel Furmedge**

UK

**Anastasia Gage**

USA

**Ettore Gagliano**

Italy

**Michael Gal**

Israel

**Linda Gallo**

Australia

**Moses Galukande**

Uganda

**Maria Gamaletsou**

UK

**Brenda Gamble**

Canada

**Esteban Gandara**

Canada

**Stuart Gansky**

USA

**Michael Gantier**

Australia

**Mingming Gao**

USA

**Vadim Gaponenko**

USA

**Samantha Garbers**

USA

**Anna Rosa Garbuglia**

Italy

**Melissa Garcia**

Australia

**Guillermo Garcia Garcia**

Mexico

**Jaime García-Mena**

Mexico

**John Gardner**

UK

**Rafael Garduno**

Canada

**Mutien-Marie Garigliany**

Belgium

**Allan Garland**

Canada

**Jennifer Garvin**

USA

**Gasim Gasim**

Sudan

**Derek Gatherer**

UK

**Charlotte Gayer-Anderson**

UK

**Zuzana Gdovinova**

Slovakia

**Lakew Gebretsadik**

Ethiopia

**Jonathan Gent**

USA

**Sara Georgiadou**

USA

**Petros Georgopoulos**

Greece

**Charles Gerardo**

USA

**Maria Gerosa**

Italy

**Robyn Gershon**

USA

**Amir A. Ghaferi**

USA

**Kioumars Ghamkhar**

New Zealand

**Akhgar Ghassabian**

USA

**Musie Ghebremichael**

USA

**Anindya Ghosh**

India

**Christoforos Giannaki**

Cyprus

**Claudia Giannetto**

Italy

**Katherine Gibney**

Australia

**Amy Lewis Gilbert**

USA

**Hazel Gilbert**

UK

**Shawn Gilbert**

USA

**Melissa Gilkey**

USA

**Simone Gill**

USA

**Gavin Gillmore**

UK

**Andreas Gilsdorf**

Germany

**Etel Gimba**

Brazil

**Jessye Melgarejo Do Amaral Giordani**

Brazil

**Ioannis Gioulbasanis**

Greece

**Jessica Gipson**

USA

**Raphaële Girard**

France

**Melissa Gladstone**

UK

**Kevin Glaser**

USA

**Deborah Glik**

USA

**Norman Goco**

USA

**Shailendra Goel**

India

**Sarah Goetz**

USA

**Laura Goetzl**

USA

**Su Kah Goh**

Australia

**John Gohagan**

USA

**Carlos Gois**

Portugal

**Sabine Goisser**

France

**Joanna B. Goldberg**

USA

**Karen Goldstein**

USA

**Srinivas Goli**

India

**Adrian Gombart**

USA

**Alexandra Goncalves**

USA

**Jorge González Domínguez**

Germany

**Marta Gonzalez-Freire**

Spain

**Benjamin Good**

USA

**Helen Goodyear**

UK

**Ganesh Gopalakrishnan**

India

**Vanathi Gopalakrishnan**

USA

**Brett Gordon**

Australia

**James Gorham**

USA

**Yevgeniy Goryakin**

UK

**Gunnar Gothberg**

Sweden

**Rossetos Gournellis**

Greece

**Tim Grabowski**

USA

**Carmelo Graffagnino**

USA

**Stuart Grande**

USA

**Robert Grant**

UK

**William Gray**

UK

**Laurie Grealish**

Australia

**Shoshana Greenberger**

Israel

**Jesica Greene**

USA

**Andrew Greenhill**

Australia

**Alex Greenwood**

Germany

**Birgit Greiner**

Ireland

**Christine Griffin**

Ireland

**Jayne Griffith**

USA

**Sergio Grimbs**

Germany

**Justin Grobe**

USA

**Dina Grohmann**

Germany

**Allison Groves**

USA

**Alejandro Gru**

USA

**Amy Grunden**

USA

**Augustine Gubba**

South Africa

**Anne-Marie Guerguerian**

Canada

**Greg Guerin**

Australia

**Patrick Guerin**

Switzerland

**Marlon Guerrero**

USA

**Parry Guilford**

New Zealand

**Per Guldberg**

Denmark

**Maria Gullinello**

USA

**Sibel Elif Gültekin**

Turkey

**Burçak Gümüs**

Turkey

**Nalika Gunawardane**

Sri Lanka

**Claudia Gundacker**

Bahamas

**Rongjun Guo**

USA

**Xue Guo**

USA

**Zhang Guozhong**

China

**Nitin Gupta**

India

**Pallav Gupta**

India

**Rakesh Gupta**

India

**Shailvi Gupta**

USA

**Emily Guseman**

USA

**Gregory Gutierrez**

USA

**Eric Haag**

USA

**Nikolas Haass**

Australia

**Rima Habib**

Lebanon

**Hubert Hackl**

Austria

**Robert Haennel**

Canada

**Curt Hagedorn**

USA

**Amy Hagopian**

USA

**Ghulam Haider**

Pakistan

**Najmul Haider**

Bangladesh

**Demewoz Haile**

Ethiopia

**Amin Hajitou**

UK

**Joerg Hakenberg**

USA

**Mythri Halappa**

India

**S. Haldar**

India

**Jostein Halgunset**

Norway

**Linda C. Halgunseth**

USA

**Arja Halkoaho**

Finland

**Alix Hall**

Australia

**Michael Hall**

Canada

**Jeffrey Hallam**

USA

**Abdul Hamood**

USA

**Greg Hampikian**

USA

**Seung Hyun Han**

South Korea

**Yuyan Han**

USA

**David Hanauer**

USA

**Johan Hanekom**

South Africa

**Ben Hankamer**

Australia

**Bethany Hannafon**

USA

**Philippe Hantson**

Belgium

**Birgit Harbeck**

Germany

**Pia Hardelid**

UK

**Kenneth Harkin**

USA

**Arthur Harralson**

USA

**Deirdre Harrington**

UK

**Abigail Harrison**

USA

**Lisa Harrison**

USA

**Reema Harrison**

Australia

**James Hartley**

UK

**Ikuko Haruta**

Japan

**Idethia Shevon Harvey**

USA

**Yasha Hasija**

India

**Crystal Haskell-Ramsay**

UK

**Saleem Hasso**

Iraq

**Bernhard Haubold**

Germany

**Arvid Steinar Haugen**

Norway

**Radim Havelek**

Czech Republic

**Susan Hawken**

New Zealand

**Alex Haynes**

USA

**Banasri Hazra**

India

**Lin He**

China

**Jodi Heaps-Woodruff**

USA

**Mary Hearst**

USA

**Maxime Hebrard**

Japan

**Manfred Hecking**

Austria

**Craig Hedberg**

USA

**Juergen Heesemann**

Germany

**Marc Heft**

USA

**Mohammad Heidari**

USA

**Roswitha Heinrich-Weltzien**

Germany

**Ellen Henderson**

UK

**M. Patrick Henriet**

Belgium

**Nicholas Henschke**

Germany

**Georg Herget**

Germany

**Peter Herman**

USA

**Lorenzo Enrique Hernández Castellano**

Switzerland

**Julio Hernandez-Camarena**

Mexico

**Diana Hernández-Romero**

Spain

**Marion Hetherington**

UK

**David Hewitt**

USA

**Winston Hide**

UK

**Michael Hier**

Canada

**Em Higa**

UK

**Andrew Hill**

New Zealand

**Blanca Himes**

USA

**David Himmelgreen**

USA

**Leslie Hinyard**

USA

**Girish Hiremath**

USA

**Tomohisa Hirobe**

Japan

**Janet Hirst**

UK

**Damian Hodgson**

UK

**Yvonne Hodgson**

Australia

**Kathleen Hoeger**

USA

**Beatrice Hoffmann**

USA

**Oliver Hofmann**

UK

**Corinne Hohl**

Canada

**Theresa Hoke**

USA

**Martin Holland**

UK

**Clare Holleley**

Australia

**Gemma Holliday**

USA

**Jennifer Hollowell**

UK

**Edward J. Hollox**

UK

**Ulrich Honemeyer**

United Arab Emirates

**Rodney Honeycutt**

USA

**Peiying Hong**

Saudi Arabia

**Steven Hong**

USA

**Maurits Hoogerwerf**

The Netherlands

**John Hopkins**

USA

**Pia Horvat**

UK

**Md. Ismail Hossain**

Bangladesh

**Bob Hou**

USA

**Jianlin Hou**

China

**Liping Hou**

USA

**John House**

Australia

**Amy Houtrow**

USA

**Daniel Howard**

USA

**Amy Howell**

USA

**Cathrine Hoyo**

USA

**Juan Hoyos**

Spain

**Ching-Liang Hsieh**

Taiwan

**Kevin Hsu**

Taiwan

**Jianzhong Hu**

USA

**Minggen Hu**

China

**Yizhou Hu**

Finland

**I-Chueh Huang**

USA

**Jinling Huang**

USA

**Lei Huang**

USA

**Tania Huedo-Medina**

USA

**Gail Hughes**

South Africa

**Joel Hughes**

USA

**Mah Siau Hui**

Malaysia

**Robbert Huijsman**

The Netherlands

**Debbie Humphries**

USA

**Catherine Hunter**

USA

**Samuel Huntley**

USA

**Daniel Huson**

Germany

**Steve Hutcheson**

USA

**Stephen William Hutchins**

UK

**Wonmuk Hwang**

USA

**Wayne Hynes**

UK

**Bede Ibe**

Nigeria

**Ogochukwu Ibeh**

Nigeria

**Jamaiah Ibrahim**

Malaysia

**Scott B. Ickes**

USA

**Masahide Ikeguchi**

Japan

**Hisao Imai**

Japan

**Mohammad Zafar Imam**

Bangladesh

**Shinsaku Imashuku**

Japan

**Aamer Imdad**

USA

**Francesco Imperi**

Italy

**Umit Ubeyt Inan**

Turkey

**Claudia Infante**

Mexico

**Roger Ingham**

UK

**Evan Ingley**

Australia

**Maia Ingram**

USA

**Alastair Innes**

UK

**Margaret Ip**

Hong Kong

**Rizwan Iqbal**

Pakistan

**Michael Iroezindu**

Nigeria

**Jennifer Irwin**

Canada

**Yasuko Ishida**

USA

**Kubilay Isik**

Turkey

**Meltem Isikgoz Tasbakan**

Turkey

**Michael Ison**

USA

**Robert Issenman**

Canada

**Munenari Itoh**

Japan

**Jaroslaw Jacak**

Austria

**Yves Jackson**

Switzerland

**Mario Jacques**

Canada

**Michel Jaffrin**

France

**Jonine Jancey**

Australia

**Benjamas Janchawee**

Thailand

**Stefan Janecek**

Slovakia

**Piotr Janicki**

USA

**Ingmar Janse**

The Netherlands

**Bettina Jansen-Schulz**

Germany

**Lauren Jantzie**

USA

**Anna Jarchow-Macdonald**

UK

**Joseph Jarvis**

South Africa

**Marjan Javanbakht**

USA

**Sara Javanparast**

Australia

**Mohammed Jawad**

UK

**Rohan Jayasuriya**

Australia

**Paul Jedlicka**

USA

**Meghan Jeffres**

USA

**Pratap Jena**

India

**Frank Jenkins**

USA

**Helen Jenkins**

USA

**Michelle Jetha**

Canada

**Yanjie Jia**

China

**Chen Jiajia**

China

**Guoqian Jiang**

USA

**Ning Jiang**

Germany

**Qingping Jiang**

China

**Anja Joachim**

Austria

**Michael Jöbges**

Germany

**Liz Joekes**

UK

**Michel Joffres**

Canada

**Jesper Johannesen**

Denmark

**Simone Johner**

Germany

**Eric Johnson**

USA

**Jonas Johnson**

USA

**Rebecca Johnson**

Australia

**Sarah Johnson**

USA

**Teresa Johnson**

USA

**Timothy Johnson**

USA

**Christine Johnston**

USA

**Janice Johnston**

Hong Kong

**Suzanne Jolley**

UK

**Philippe Joly**

France

**Andrew Jones**

UK

**Lynne Jones**

USA

**Makoto Jones**

USA

**Martina Jones**

Australia

**Randall Jorgensen**

USA

**Matthew Jose**

Australia

**John Joska**

South Africa

**Eric Judd**

USA

**Howard Judelson**

USA

**Freimut Juengling**

Switzerland

**Jennifer Juno**

Canada

**Sian K. Smith**

Australia

**Haytham Kaafarani**

USA

**Ashley Kable**

Australia

**Achille Kabore**

USA

**Chodziwadziwa Kabudula**

South Africa

**Nii-Kabu Kabutey**

USA

**Roland Kaddoum**

Lebanon

**Tamilarasu Kadhiravan**

India

**Kamran Kadkhoda**

Canada

**Kostantinos Kafetsios**

Greece

**Sherif Kafrawy**

Saudi Arabia

**Hristos Kaimakliotis**

USA

**Chikara Kaito**

Japan

**Yasuharu Kaizaki**

Japan

**Ignatius Kakande**

Rwanda

**Hironaga Kakoi**

Japan

**Kaarthigeyan Kalaniti**

Canada

**Nikolaos Kalinderis**

Greece

**Manoj Kalita**

India

**Krishnamani Kalpathi**

India

**Kameljit Kalsi**

UK

**Sunil Kamat**

USA

**Erdinc Kamer**

Turkey

**Peter Kamerman**

South Africa

**Kenya Kamimura**

Japan

**Mona Kanaan**

UK

**Gokul Vignesh Kanda Swamy**

UK

**Mahesh Kandasamy**

India

**Chitra Kannabiran**

India

**M. Kannan**

India

**Astrid Kännaste**

UK

**Sashi Kant**

USA

**Amer Karam**

USA

**Ece Karatan**

USA

**Peter Kardos**

UK

**Slim Karkar**

USA

**Rajendra Karkee**

Nepal

**James Karlowsky**

Canada

**S. Ananth Karumanchi**

USA

**Panduka Karunanayake**

Sri Lanka

**Peter Helmuth Karuso**

Australia

**John Kasckow**

USA

**Saba Kassim**

UK

**Maria Katapodi**

Switzerland

**Gohei Kato**

Japan

**Ioannis Katsarolis**

Greece

**Kendra Kattelmann**

USA

**David Katz**

USA

**Pankaj Kaul**

UK

**Paramjit Kaur**

India

**Mitsuhiro Kawano**

Japan

**Michael Kawooya**

Uganda

**Reem Kayyali**

UK

**Abby Kazley**

USA

**Guan-Ming Ke**

Taiwan

**Hannah Keage**

Australia

**Moritz Kebschull**

Germany

**Mark Keegan**

USA

**Philip Keeley**

UK

**Marc Keirse**

Australia

**Jonathan Keith**

Australia

**Hillary Kelly**

USA

**Shona Kelly**

UK

**Jamie Kennedy**

USA

**Lindsey Kennedy**

USA

**May G. Kennedy**

USA

**Sean Kennedy**

Australia

**Amy Kenter**

USA

**Deanna Kepka**

USA

**Jocelyn Kernot**

Australia

**Chandrasekhar Kesavan**

USA

**Serhat Keser**

Turkey

**Robin Ketteler**

UK

**Maryam Khalili**

USA

**Ejaz Khan**

Pakistan

**Jawaid Ahmad Khan**

India

**Mohammad Asif Khan**

Malaysia

**Muhammad Khan**

Pakistan

**Rafeeq Khan**

Pakistan

**Shah Alam Khan**

Oman

**Gulam Khandaker**

Australia

**Hootan Khatami**

USA

**Mahnaz Khatiban**

Iran

**Mozafar Khazaei**

Iran

**Sok Kean Khoo**

USA

**Kim Kiely**

Australia

**Yibeltal Kiflie**

USA

**Kwang-Sik Kim**

USA

**Seok-Won Kim**

Japan

**Seon-Young Kim**

South Korea

**Seung-Ki Kim**

South Korea

**Martin Kimanya**

Tanzania

**Pamela Kimeto**

USA

**Brian King**

USA

**Tania King**

New Zealand

**Michael Kingsley**

Australia

**Serafim Kiriakidis**

UK

**Edwin Kirk**

Australia

**Philipp Kirsch**

Australia

**Shin-Ichiro Kitajiri**

Japan

**Alan Kitchen**

UK

**Olivia Kituuka**

Uganda

**Jeffrey Klausner**

USA

**Anita Klein**

USA

**Samuel Klempner**

USA

**Gert Klug**

Austria

**Joshua Knowles**

USA

**Bryan Knuckley**

USA

**Won-Jung Koh**

South Korea

**Filip Kolar**

Czech Republic

**Thomas Kolon**

USA

**Linglong Kong**

Canada

**Pornpimol Kongtip**

Thailand

**Evangelos Kontopantelis**

UK

**Stephen Kopecky**

USA

**Mustafa Koplay**

Turkey

**Darren Korbie**

Australia

**Peter Kornprat**

Austria

**Nicole Koropatkin**

USA

**Wim Kort**

The Netherlands

**Dhananjay Kotasthane**

India

**Ramesh Kothari**

India

**Vijay Kothari**

India

**Grigorios Kotronoulas**

UK

**Paul Kotzbauer**

USA

**Nicholas George Kounis**

Greece

**Argyro Krase**

Greece

**Matthew D. Krasowski**

USA

**Robert Kraus**

Germany

**Nancy Kressin**

USA

**Jimmie Kristensson**

Sweden

**Alfgeir Logi Kristjansson**

USA

**Vicki Kristman**

Canada

**Kurt Kroenke**

USA

**Andreas Kronbichler**

Austria

**Janet Krska**

UK

**Alexey Kryukov**

Russian Federation

**Suresh Kuchipudi**

USA

**Abhay Kudale**

India

**Anne Sebert Kuhlmann**

USA

**Pavel Kuksa**

USA

**Shunichi Kumakura**

Japan

**Dharmendra Kumar**

India

**Nishant Kumar**

India

**Atsushi Kurabayashi**

Japan

**Biji Kurien**

USA

**Lesia Kurlak**

UK

**Lalith Kuruppuarachchi**

Sri Lanka

**Tetsuro Kusaba**

USA

**Shigeru Kusagawa**

Japan

**Mary Kwaan**

USA

**Kwabena Kwakye**

Ghana

**Naa Oyo Kwate**

USA

**Cannas Kwok**

Australia

**Salil Lachke**

USA

**Rollin Laetitia**

France

**Chih-Cheng Lai**

Taiwan

**Jimmy Lai**

Hong Kong

**Hooi Lai Seong**

Malaysia

**Girdhari Lal**

India

**Rup Lal**

India

**Jorge Lalucat**

Spain

**Aart Lammers**

The Netherlands

**Stacey Lance**

USA

**Přemysl Landa**

Czech Republic

**James Lane**

USA

**Richard Lange**

USA

**David Langenberger**

Germany

**Rebecca Langford**

UK

**Horst-Christian Langowski**

Germany

**Muriel H. Larauche**

USA

**Emma Larkin**

USA

**Carsten Schade Larsen**

Denmark

**David Larsen**

USA

**Heidi Larson**

UK

**Janet Larson**

USA

**Dennis Laryea**

Ghana

**Tim Lau**

Canada

**M. Barton Laws**

USA

**Phuc Le**

USA

**Paul Le Tissier**

UK

**Tricia Leahey**

USA

**Vijittra Leardkamolkarn**

Thailand

**David Lebeaux**

France

**Tomasz Lech**

Poland

**John Lednicky**

USA

**Byeong Chun Lee**

South Korea

**Eun Bong Lee**

South Korea

**France Eun-Hyung Lee**

USA

**Gun Woo Lee**

South Korea

**Jason Lee**

Singapore

**Keon-Young Lee**

South Korea

**Elizabeth Lefler**

USA

**Catherine C. Legrand**

Belgium

**Venla Lehti**

Finland

**Liancheng Lei**

China

**Jorge Humberto Leitão**

Portugal

**Robert Leman**

USA

**Seblewengel Lemma**

Ethiopia

**Rosa Lemmens-Gruber**

Austria

**Rebecca Leonard**

USA

**Aymeric Leray**

France

**Simmons Lessell**

USA

**Leanne Lester**

Australia

**Philipp Leucht**

USA

**Loh-Shan Leung**

USA

**Susanna Lewerin**

Sweden

**Stephen Lewis**

UK

**Bing-Zhi Li**

China

**Gui Li**

China

**Jihong Li**

USA

**Jinghong Li**

USA

**Lin Li**

USA

**Shou-Hsien Li**

Taiwan

**Xiang Li**

USA

**Xiu-Qing Li**

Canada

**Yupeng Li**

USA

**Vassilios Liakopoulos**

Greece

**Limin Liao**

China

**Marc Libault**

USA

**Brett Lidbury**

Australia

**Uwe G. Liebert**

Germany

**Gabriele Lignani**

UK

**Rebbecca Lilley**

New Zealand

**Rebecca Lim**

Australia

**Chao-Hsu Lin**

Taiwan

**David M. Lin**

USA

**Tao Lin**

USA

**Yan Lin**

USA

**Tülay Lindberg**

Sweden

**Jennifer Linde**

USA

**Jiang Lin-Hua**

UK

**Robert Liston**

Canada

**Chia-Chyi Liu**

Taiwan

**Jun Liu**

China

**Ko-Jiunn Liu**

Taiwan

**Ming-Tsan Liu**

Taiwan

**Sumei Liu**

USA

**Yang Liu**

USA

**Zhongyuan Liu**

USA

**Y. L. Lo**

Singapore

**Paula Lobato De Faria**

Portugal

**Susan J. Loeb**

USA

**Adrian Loerbroks**

Germany

**Loretta Loftus**

USA

**Jessica Logan**

USA

**M. Cynthia Logsdon**

USA

**Alayna Loiselle**

USA

**Ellen Løkkegaard**

Denmark

**Thomas London**

USA

**Marie Londrigan**

USA

**Richard Long**

Canada

**Igor Loniewski**

Poland

**Susana Lopes**

Portugal

**Thiago Lopes**

Australia

**Everardo López-Romero**

Mexico

**Matthew Lorincz**

Canada

**Monicque Lorist**

The Netherlands

**Marianne Løvstad**

Norway

**Julia Lowe**

Canada

**Martin Löwer**

Germany

**Xinghua Lu**

USA

**Sam Lubner**

USA

**Tim Luckett**

Australia

**Aneta Luczkiewicz**

Poland

**Peter Lüdemann**

Germany

**Ndiko Ludidi**

South Africa

**Marjolein Lugtenberg**

The Netherlands

**Bohdan Luhovyy**

Canada

**Ochiba Lukandu**

Kenya

**Vimoksalehi Lukoschek**

Australia

**Pipat Luksam**

Thailand

**Ma Luo**

Canada

**Mark Lurie**

USA

**Jaber Lyahyai**

Morocco

**Jan Lycke**

Sweden

**Alan Lyles**

USA

**Catherine Lynch**

USA

**Qin Ma**

USA

**Xuejun Ma**

China

**Zhiwei Ma**

USA

**Peter Mace**

New Zealand

**Mark Macek**

USA

**António Machado**

Ecuador

**Lisa Mackenzie**

Australia

**Karl Madaras-Kelly**

USA

**Franklin Maddux**

USA

**Nyovani Madise**

UK

**Yoshiaki Maeda**

Japan

**Yoshihiko Maehara**

Japan

**Susan Magasi**

USA

**Pascal Magnussen**

Denmark

**Nancy Mah**

Germany

**Aba Mahamat**

French Guiana

**Bidhubhusan Mahapatra**

India

**Elizabeth Maher**

USA

**Shehrin Mahmood**

Bangladesh

**Hanan Mahmoud**

Egypt

**Bani-Yaghoub Majid**

USA

**Mtebe Majigo**

Tanzania

**Fredrick Makumbi**

Uganda

**Pasqualino Malandrino**

Italy

**Hoda Malaty**

USA

**Carl Malchoff**

USA

**Ayesha Malik**

Pakistan

**Elfatih Malik**

Sudan

**Thriveen Sankar Chittoor Mana**

USA

**Rita Manco**

Belgium

**Peter Mancuso**

USA

**Diptasri Mandal**

USA

**Jess Mandel**

USA

**Kate Mandeville**

UK

**Andrea Manetti**

Italy

**Subramani Mani**

USA

**Bruce Mann**

Australia

**Jim Manos**

Australia

**Joaquin Manrique**

Spain

**Kerry Mansell**

Canada

**Avril Mansfield**

Canada

**Nadia Mantovani**

UK

**Luigi Marchionni**

USA

**Maria Marco**

USA

**Johanna Maree**

South Africa

**Claudia Marhuenda**

Spain

**Karen Marienau**

USA

**Michel Marina**

Spain

**Gian Mariottini**

Italy

**Konstantinos Markou**

Greece

**Wojciech Marlicz**

Poland

**Michael Marmor**

USA

**Alexandre Marra**

Brazil

**Bernadette P. Marriott**

USA

**Ian Marschner**

Australia

**Stuart Marshall**

Australia

**Pier Luigi Martelli**

Italy

**Sean Martin**

Australia

**Richard Martinello**

USA

**Allyson Martinez**

USA

**Marc A. Marti-Renom**

Spain

**Yousef Marwan**

Kuwait

**David Allan Marzano**

USA

**Enrica Marzola**

Italy

**Linnet Masese**

Kenya

**Khathutshelo Mashige**

South Africa

**Simona Masiero**

Italy

**Oteh Maskon**

Malaysia

**Saber Masmoudi**

Tunisia

**Robin Mason**

Canada

**Sébastien Massier**

France

**Gift Matope**

Zimbabwe

**Joseph Matovu**

Uganda

**Paul Matthews**

USA

**Slade Matthews**

Australia

**Christina Matz-Costa**

USA

**Thais Mauad**

Brazil

**Marzorati Mauro**

Italy

**Laurent Mauvieux**

France

**Kenneth Mavor**

UK

**Maureen Mayhew**

Canada

**Darren Mays**

USA

**Leonard Mboera**

Tanzania

**Erasto Mbugi**

Tanzania

**Elizabeth McAninch**

USA

**Fiona McCarthy**

USA

**Robert McCarthy**

USA

**Affette McCaw-Binns**

Jamaica

**Scott McComb**

Switzerland

**Gregory McCracken**

Canada

**Paul McDaniel**

USA

**Christine McDonough**

USA

**Richard McGee**

USA

**Jane McGowan**

USA

**Alastair McGregor**

UK

**Meredith McIntyre**

Australia

**Ruth McKee**

UK

**Martin McKibbin**

UK

**John McLachlan**

UK

**Sarah McLachlan**

UK

**Mary Ann McLane**

USA

**William McLellan**

USA

**Alex D. McMahon**

UK

**Matthew McMillin**

USA

**Kevin McMullen**

USA

**Daniel McWilliams**

UK

**Daniel Meara**

USA

**Hiren Mehta**

USA

**Saurabh Mehta**

USA

**Supriya Mehta**

USA

**Zeleke Mekonnen**

Ethiopia

**Kathleen Melanson**

USA

**Joshua Mell**

USA

**Ann Melvin**

USA

**Lia Menasce**

UK

**Fernando Mendez**

USA

**Elismauro Mendonça**

Brazil

**Fanyin Meng**

USA

**Heather Menzies**

USA

**Shahin Merat**

Iran

**Anwar Merchant**

USA

**Neil Merrett**

Australia

**Sharon Messenger**

USA

**Anna Meyer-Weitz**

South Africa

**Gerben Meynen**

The Netherlands

**Aliapsha Meysamie**

Iran

**Osaro Mgbere**

USA

**Kurt Michael**

USA

**Ioannis Michopoulos**

Greece

**Suresh Mickymaray**

Saudi Arabia

**James Millard**

UK

**Jeff Milleman**

USA

**Irina Mindlis**

USA

**Maria Miragaia**

Portugal

**Goksel Misirli**

UK

**Ralph Mistlberger**

Canada

**Caroline Mitchell**

USA

**Xiulei Mo**

USA

**Vernon Mochache**

UK

**Eduardo Moffa**

Brazil

**Jamaludin Mohamad**

Malaysia

**Mansour Mohamadzadeh**

USA

**Amir Mohammad**

USA

**Fouad Mohammad**

Iraq

**Ghorbanali Mohammadi**

Iran

**Osama Mohammed**

Saudi Arabia

**Vidyarani Mohankumar**

India

**David Mohr**

USA

**Max Moldovan**

Australia

**Barbara Molesini**

Italy

**Sophie Molia**

Mali

**Gustavo Molina**

Argentina

**Carmen A. Molina-Torres**

Mexico

**Kathryn Momary**

USA

**Nitish Mondal**

India

**Marc Monot**

France

**Todd Monroe**

USA

**Francesca Montagnani**

Italy

**Carl Montague**

South Africa

**Jesus Montero-Marin**

Spain

**Gustavo Monti**

Chile

**Thomas Mooe**

Sweden

**Troy Moon**

USA

**Catrin Moore**

UK

**Melinda Moore**

USA

**Meghan Moran**

USA

**Nyambura Moremi**

Tanzania

**Burkhard Morgenstern**

Germany

**Kazuhiro Morishita**

Japan

**Chigusa Morizane**

Japan

**Laura Moro**

Spain

**Glen Morrell**

USA

**J. Bradley Morris**

USA

**John Morris**

USA

**Nancy Morris**

USA

**Lynn Morrison**

USA

**Daniel Morton**

USA

**Mohammad Motamedi**

Iran

**Davide Motta**

Italy

**Varvara Mouchtouri**

Greece

**Panagiotis Moulos**

Greece

**Johann Mourier**

France

**Mwifadhi Mrisho**

Tanzania

**Stephen Mshana**

Tanzania

**Hiltrud Muhle**

Germany

**Andrew Mujugira**

Uganda

**Souryadeep Mukherjee**

India

**Vincent Mukkada**

USA

**Victor Mulanovich**

USA

**Andargachew Mulu**

Ethiopia

**Caroline Mulvaney**

UK

**Matthew Mulvey**

USA

**Nicola Mumoli**

Italy

**César Munayco**

USA

**Shama Munim**

Pakistan

**Maria Munne**

Argentina

**Violeta Muñoz-Fuentes**

Spain

**Ichiro Murakami**

Japan

**Francis Muregi**

Kenya

**Andrea Murina**

USA

**Sean Murphy**

USA

**Jayaseelan Murugaiyan**

Germany

**Claudio Musetti**

Italy

**David Musick**

USA

**Charles Musselwhite**

UK

**Maria Carolina Martins Mussi**

Brazil

**Tshilidzi Muthivhi**

South Africa

**Veerappan Muthukkaruppan**

India

**Adamson Muula**

Malawi

**Marshal Mweu**

Kenya

**Garry Myers**

Australia

**Matti Myllykoski**

Finland

**Karthikeyan Mythreye**

USA

**Kurt G. Naber**

Germany

**Michael Nadorff**

USA

**Ramesh Nagarajappa**

India

**Hampapathalu Adimurthy Nagarajaram**

India

**Olivier Naggara**

France

**Manju Nagpal**

India

**Zoltan Nagy**

Hungary

**Ramzi Nahhas**

USA

**Girish Nair**

Canada

**Meera Nair**

USA

**Motohiro Nakajima**

USA

**Masayuki Nakamichi**

Japan

**Junichi Nakamura**

Japan

**Bejoy Nambiar**

Malawi

**Manasi Nandi**

UK

**Francis Nano**

Canada

**Georgios Naros**

Germany

**Muhammad Naseer**

Pakistan

**Jeyakumar Natarajan**

India

**K. Natarajaseenivasan**

India

**Christopher Naugler**

Canada

**S. M. Naushad**

India

**Gerardo Nava**

USA

**Jesús Navarro**

Spain

**Carlos Navarro Colorado**

USA

**Sonia Navas-Martin**

USA

**Prasunpriya Nayak**

India

**Gaurang Nazar**

India

**Abd Jabar Nazimi**

Malaysia

**Khm Nazmul H. Nazir**

Bangladesh

**Mzikazi Nduna**

South Africa

**Anne Victoria Neale**

USA

**Pradeep Negi**

India

**Praveen Nekkar Rao**

Canada

**David Nelson**

USA

**Michael Nemergut**

USA

**Ujjwal Neogi**

Sweden

**Irene Newsham**

USA

**William Newsholme**

UK

**Ka-Lok Ng**

Taiwan

**Joseph Nguta**

Kenya

**Lynne Nguyen**

USA

**Maribeth Nicholson**

USA

**Henrik Nielsen**

Denmark

**Martin Nielsen**

USA

**Stig Lønberg Nielsen**

Denmark

**Emily Niemeyer**

USA

**Albert Nienhaus**

Germany

**Nathan Nieto**

USA

**Wiktor Niewiadomski**

Poland

**Dimitrios Nikolopoulos**

Greece

**Milena Nikolova**

Bulgaria

**Daisuke Nishi**

Japan

**Hiroshi Nishimune**

USA

**Dorothea Nitsch**

UK

**Theo Niyonsenga**

Australia

**Damir Nizamutdinov**

USA

**Fidelis Njokanma**

Nigeria

**Edward Nketiah-Amponsah**

Ghana

**Soori Nnko**

Tanzania

**James Noble**

USA

**Catherine Nock**

Australia

**Hidetaka Noma**

Japan

**Cristina Noriega**

Spain

**Richard Norman**

Australia

**Kathleen Norr**

USA

**Laura Norris**

USA

**Gabriela Nosalova**

Slovakia

**Jean Jacques Noubiap**

Cameroon

**Masafumi Nozawa**

Japan

**Alan Nugent**

USA

**William Nunery**

USA

**Rolf Nüsing**

Germany

**Sabina Nuti**

Italy

**Samuel Robert Nyman**

UK

**Jennifer Oates**

Australia

**Joshua Ayoola Obaleye**

Nigeria

Gerald Obermair

Austria

**Eivor Oborn**

UK

**Michael O’Callaghan**

Australia

**Maria T. Ochoa**

USA

**Catherine O’Connor**

Australia

**Sophie Octavia**

Australia

**Funlayo Odejinmi**

UK

**Clifford Odimegwu**

South Africa

**Kieran O’Doherty**

Canada

**Ernest Oertli**

USA

**Rei Ogawa**

Japan

**Clodagh O’Gorman**

Ireland

**Tinuade Ogunlesi**

Nigeria

**Kazuhiro Ohwaki**

Japan

**Chika Okafor**

USA

**Denise O’Keefe**

USA

**Charles Okoli**

Nigeria

**Ijeoma Okoronkwo**

Nigeria

**Ikechi Okpechi**

South Africa

**Ogi Okwumabua**

USA

**Bolanle Ola**

Nigeria

**Baldo Oliva**

Spain

**Rene Olivares-Navarrete**

USA

**Kent Olson**

USA

**Robert Olson**

Canada

**Gabrielle O’Malley**

USA

**Frankline Magaki Onchiri**

Kenya

**Macaulay Onuigbo**

USA

**Vincent Onywera**

Kenya

**Joukje Oosterman**

The Netherlands

**Maaike Oosterveer**

The Netherlands

**Antone R. Opekun**

USA

**Japheth Opintan**

Ghana

**David Ornelles**

USA

**Heather Orom**

USA

**J. Miguel Ortega**

Brazil

**Javier Ortego**

Spain

**Kevin O’Shaughnessy**

UK

**Kiyoko Oshima**

USA

**Babatunde Osinaike**

Nigeria

**Carla Osiowy**

Canada

**Em Osoro**

Kenya

**Robert Ostfeld**

USA

**Julie Ostrander**

USA

**Rennolds Ostrom**

USA

**Larissa Otero**

Peru

**Ahmad Oulmouden**

France

**Foluso Owotade**

Nigeria

**Wellington Oyibo**

Nigeria

**Carly Pacanowski**

USA

**Joel Pachter**

USA

**Jasdeep Padaria**

India

**Kalliopi Pafili**

Greece

**Amanda Page**

Australia

**Timothy Page**

USA

**Varadraj Pai**

India

**Eric Pailhoux**

France

**Fauzia Paize**

UK

**Martin Palamuleni**

South Africa

**Pamela Palasanthiran**

Australia

**Michael Palese**

USA

**Lawrence Palinkas**

USA

**Prasit Palittapongarnpim**

Thailand

**Ruth Palmer**

Sweden

**Daniela Palomba**

Italy

**Enzo Palombo**

Australia

**Vincent Palusci**

USA

**Xiaoyue Pan**

USA

**Zenggang Pan**

USA

**Aditya K. Panda**

India

**Anand Pandey**

India

**Rakesh Pandey**

India

**Nikolaos Pandis**

Switzerland

**Tiziana Pandolfini**

Italy

**Vijayapandi Pandy**

Malaysia

**Jia-Min Pang**

UK

**Jürgen Pannek**

Switzerland

**Eric Pante**

France

**Annalisa Pantosti**

Italy

**Nikolaos Papanas**

Greece

**Athanasia Papazafiropoulou**

Greece

**Dimitrios Papazoglou**

Greece

**Jesse Papenburg**

Canada

**Bongkyun Park**

South Korea

**R. David Parker**

USA

**Donovan Parks**

Canada

**Priya Parmar**

New Zealand

**Eric Parmentier**

Belgium

**Abhishek Parolia**

Malaysia

**Paolo Pasquali**

Italy

**Stefano Passanisi**

Italy

**Bhoomika Patel**

India

**Nilesh Patel**

Kenya

**Sachin Patel**

USA

**Shashikant Patne**

India

**Robert Patton**

UK

**Paisit Paueksakon**

USA

**Jaishree Paul**

India

**Harald Paulsen**

Germany

**Mary Ellen Pavone**

USA

**Justin Peacock**

USA

**Prashanth Peddi**

USA

**Torstein Pedersen**

Norway

**Gökhan Pekel**

Turkey

**Marco Pelin**

Italy

**Lee Peng**

USA

**Lingtao Peng**

USA

**Anthony Penington**

Australia

**Suprasanna Penna**

India

**Alan Pepper**

USA

**Massimo Perachino**

Italy

**Ana Pereira Cavaleiro**

Portugal

**Jennifer Perera**

Sri Lanka

**Isabel Pérez-Olmos**

Colombia

**Thorsten Perl**

Germany

**Fabien Pernot**

France

**Lin Perry**

Australia

**Adam Persky**

USA

**Sarah Peters**

UK

**Gregory Peterson**

Australia

**Andrew Petrosoniak**

Canada

**Snezana Petrovic**

USA

**Emil Petrusa**

USA

**Stefano Petti**

Italy

**Vlad Petyuk**

USA

**David Phalen**

Australia

**Jack Phan**

USA

**Jennifer Pharr**

USA

**Jean Marc Phelip**

France

**Koshy Philip**

Malaysia

**Caleb Phillips**

USA

**J. Craig Phillips**

Canada

**Patrizia Picerno**

Italy

**Kim Picozzi**

UK

**Robert Pilarski**

USA

**June Pilcher**

USA

**Stefano Pileri**

Italy

**Gustavo Duarte Pimentel**

Brazil

**Marilia Rita Pinzone**

Italy

**Antonio Piralla**

Italy

**Katarzyna Pisanski**

Canada

**Mauro Pittiruti**

Italy

**Martin Ward Platt**

UK

**Jodie Plumert**

USA

**Laura Jean Podewils**

USA

**Monika Pogorzelska-Maziarz**

USA

**Eugenie Poh**

Singapore

**Hendrik Poinar**

Canada

**Chiara Poletto**

France

**Gregor Pollach**

Malawi

**David Poller**

UK

**Estella Poloni**

Switzerland

**Jeanine Pommier**

France

**Andrea Poretti**

USA

**Maria Grazia Porpora**

Italy

**Paola Postacchini**

Italy

**Gabriela Postolache**

Portugal

**Ilyas Potamitis**

Greece

**James Powes**

USA

**Bunny Pozehl**

USA

**Amit Prasad**

India

**Michael Prentice**

Ireland

**Marian Price-Carter**

New Zealand

**James Prior**

UK

**Charalampos Proestos**

Greece

**Christin Pruett**

USA

**Steven Pryjmachuk**

UK

**Eve Puffer**

USA

**Rajpal S. Punia**

India

**Mary Ellen Purkis**

Canada

**Birgit Puschner**

USA

**Chris Pyke**

Australia

**Paula A. Quatromoni**

USA

**Jennifer Quinlan**

USA

**Miguel Quinones-Mateu**

USA

**Jennifer Quint**

UK

**Peter Rabinowitz**

USA

**Ravi Radhakrishnan**

USA

**Peter Radsel**

Slovenia

**Malathi Raghavan**

Canada

**Siavash Rahimi**

UK

**A. K. M. Anisur Rahman**

Bangladesh

**Atiar Rahman**

Bangladesh

**Khondaker Miraz Rahman**

UK

**Iyad Rahwan**

United Arab Emirates

**Senaka Rajapakse**

Sri Lanka

**Kanniah Rajasekaran**

USA

**Geeta Ram**

USA

**Jayaparavathy Raman**

India

**Divya Ramesh**

USA

**Sandra Ramirez**

Canada

**Mariana Ramos**

Peru

**Asha Rani**

USA

**Ravi Ranjan**

USA

**Shripada Rao**

Australia

**Kilian Rapp**

Germany

**Adam Rash**

UK

**Shariq Rashid**

India

**David Rasko**

USA

**Trishna Rathod**

UK

**Elena Ratschen**

UK

**Anders Raustorp**

Sweden

**V. Raveenthiran**

India

**Pallab Ray**

India

**Duria Rayis**

Egypt

**Simon Rayner**

Norway

**William Raynovich**

USA

**Wantana Reanmongkol**

Thailand

**Amanda Rebar**

Australia

**Terri Rebmann**

USA

**Lawrence Recht**

USA

**Dinah Reddihough**

Australia

**Yuvaram Reddy**

USA

**Niamh Redmond**

UK

**Sean Redmond**

USA

**Maggie Redshaw**

UK

**Krishna Regmi**

UK

**Michael Reichel**

Australia

**Marvin Reid**

Jamaica

**Angela Reiersen**

USA

**Ulrike Reiss**

USA

**Chan Renee**

China

**Yeong- Renn Chen**

USA

**Lothar Resch**

Canada

**Fariba Rezaee**

USA

**Simone Ribero**

UK

**Luisel Rickssanti**

USA

**Kate Ridley**

Australia

**Irmgard Riedmaier**

Germany

**William Riley**

USA

**Bertus Rima**

UK

**Fernando Rivera-Cabrera**

Mexico

**Farhana Rizwan**

Bangladesh

**Shawn Robbins**

Canada

**Stanley Robboy**

USA

**Daniel Roberts**

USA

**Helen Roberts**

UK

**Karen Robinson**

UK

**Roger Rochat**

USA

**Rosa Rodriguez-Monguio**

USA

**Leigh Simon Roeger**

Australia

**Ana Rojas**

USA

**Hanna Rokita**

Poland

**Steve Rolfe**

UK

**Andrea Romani**

USA

**Paolo Romano**

Italy

**Ruben Roman-Ramos**

Mexico

**Bryan Romito**

USA

**Marta Rondon**

Peru

**Clara Suemi Rosa**

Brazil

**Laura Rosella**

Canada

**Mark Rosenfield**

USA

**Matthew Rose-Zerilli**

UK

**Usman Roshan**

USA

**Matt Ross**

USA

**Pablo Ross**

USA

**Liza Rovniak**

USA

**Andres M. Rubiano**

Colombia

**Michael Rubin**

USA

**Sara Rubinelli**

Sweden

**Enrique Ruiz De Gopegui**

Spain

**Ana Ruiz-Casado**

Spain

**Charles Rupprecht**

USA

**Scott Russell**

USA

**Sebastian Russo**

Germany

**Ligia Rusu**

Romania

**Joachim Rychly**

Germany

**V. S. Periasamy**

Saudi Arabia

**Steffanie Sabbaj**

USA

**Aly Saber**

Egypt

**Lora Sabin**

USA

**Binod Sah**

Nepal

**Sanjib Saha**

Bangladesh

**Sarmistha Saha**

India

**Sibu Saha**

USA

**Daisy Sahoo**

USA

**Sarmad Said**

USA

**Hassan Saidi**

Kenya

**Eric Saillant**

USA

**Wataru Saito**

Japan

**Seiichi Sakamoto**

Japan

**Cristiano Salata**

Italy

**Massimiliano Salati**

Italy

**Gustavo Adolfo Salazar**

South Africa

**Amir Saleem**

Taiwan

**Jason Salemi**

USA

**Cassandra Salgado**

USA

**Cristina Salibay**

Philippines

**Gregory Salinas**

USA

**Haroon Saloojee**

South Africa

**Sonja Sälzer**

Germany

**Krishnaswamy Sampathkumar**

India

**Janneke N. Samsom**

The Netherlands

**Abdallah Samy**

USA

**Ana Sanchez**

Canada

**Susan Sanchez**

USA

**Amarilis Sanchez-Valle**

USA

**Arun Kumar Sangaiah**

India

**Vidya Sankar**

USA

**Senthil Kumar Sankareswaran**

India

**Gaetano Santulli**

USA

**Surasak Saokaew**

Thailand

**Joseph Adusei Sarkodie**

Ghana

**Latha Satish**

USA

**Michael Satlin**

USA

**Hiroaki Satoh**

Japan

**Sarah Satola**

USA

**Anji Satoskar**

USA

**Travis Saunders**

Canada

**Azadeh Sayarifard**

Iran

**Michael Schachter**

UK

**Daniel Schachtman**

USA

**Georgia Schafer**

South Africa

**Rene Schalk**

The Netherlands

**Rob M. H. Schaub**

The Netherlands

**Phillip Scheinberg**

USA

**Richard H. Scheuermann**

USA

**Vanessa Schick**

USA

**Frank Schiedel**

Germany

**Karen Schlauch**

USA

**Jens Schlossmann**

Germany

**Robert Schmidt**

USA

**Eberhard Schneider**

Germany

**Dale Schoeller**

USA

**Marina Schoemaker**

The Netherlands

**Patricia Schoenlein**

USA

**Birgitte Jjm Schoenmakers**

Belgium

**Irvin Schonfeld**

USA

**Florian Schroeck**

USA

**Harry Schroeder**

USA

**Ewoud Schuit**

The Netherlands

**Thomas Schulte**

USA

**Tim Schultz**

Australia

**John Schuna**

USA

**Kenneth Schwartz**

USA

**Kevin Schwartzman**

Canada

**Katrina Scior**

UK

**David Scott**

USA

**Anna Seale**

UK

**Sarah Seaton**

UK

**Siby Sebastian**

USA

**Anne Sebert Kuhlmann**

USA

**Neil Sebire**

UK

**Sidy Seck**

Senegal

**Azman Seeni**

Malaysia

**Alissa Segal**

USA

**Abiy Seifu**

Ethiopia

**Elisabeth Seigner**

Germany

**Balaraman Sekar**

India

**Nick Sekouris**

UK

**David Sela**

USA

**Linda Selvey**

Australia

**Tuhinadri Sen**

India

**Marjanne Senekal**

South Africa

**Christian Senft**

Germany

**Sohn Seonghyang**

South Korea

**Javier Seravalli**

USA

**Joao Serodio**

Portugal

**Sriram Seshadri**

India

**Tesfaye Setegn**

Ethiopia

**Sunil Sethi**

India

**Helena Seth-Smith**

UK

**Salvatore Settineri**

Italy

**Osman Ugur Sezerman**

Turkey

**Dhvanit Shah**

USA

**Labib Sharif**

Jordan

**Radha Sharma**

India

**Shiwani Sharma**

Australia

**Peter Sharp**

Australia

**Andrew Shaw**

UK

**Lindsey Shaw**

USA

**Debebe Shaweno**

Ethiopia

**Sandra Shefelbine**

USA

**Edlira Shehu**

Germany

**Hong-Bin Shen**

China

**Kui Shen**

USA

**Li Shen**

USA

**Li-Jiuan Shen**

Taiwan

**X. Shen**

China

**Shweta Shenoy**

India

**Vicky Sheppeard**

Australia

**Rezvi Sheriff**

Sri Lanka

**Amir Sherman**

Israel

**Hemant Shewade**

India

**Lei Shi**

China

**Wenyin Shi**

USA

**Jun Shigemura**

Japan

**Keiko Shikako-Thomas**

Canada

**Rebecca Shilling**

USA

**Tomohiko Shimada**

Japan

**Yusuke Shimakawa**

France

**Margret Shirinian**

Lebanon

**Leonie Short**

Australia

**Matthew Shotwell**

USA

**Cheryl Shuman**

Canada

**Lori Shutter**

USA

**Kah-Ling Sia**

Australia

**Jason Sicklick**

USA

**Peter Sidebotham**

UK

**Avner Sidi**

USA

**Birgit Sikkema-Raddatz**

The Netherlands

**Dan-Arin Silasi**

USA

**Gaynell Simpson**

USA

**Scot Simpson**

Canada

**Alper Sinanoglu**

Turkey

**Steven Singer**

USA

**Debra Singh**

Uganda

**Illina Singh**

UK

**Kavita Singh**

USA

**Rajiv Singla**

India

**Nikolaos Sipsas**

Greece

**Enrico Siragusa**

Germany

**Chukiat Sirivichayakul**

Thailand

**Diego Sisci**

Italy

**Cindy Sit**

Hong Kong

**Petros Skapinakis**

Greece

**Erik Skarsgard**

Canada

**Margie Skeer**

USA

**Vitali Skoropad**

Russian Federation

**Stuart Slavin**

USA

**Derek Sloan**

Malawi

**Michelle Slone**

Israel

**David Smajs**

Czech Republic

**Nada Smigic**

Serbia

**Anna Jo Smith**

USA

**Patrick Smith**

USA

**Tara Smith**

USA

**Woutrina Smith**

USA

**Davida Smyth**

USA

**Ban Leong Sng**

Singapore

**Andrew Snow**

USA

**Marcelo Soares**

Brazil

**Mario Soares**

Australia

**Kathryn Sobek**

USA

**S. M. Afzal Sohaib**

UK

**Sukhwinder Singh Sohal**

Australia

**Marylou V. Solbrig**

USA

**Mary Lou Sole**

USA

**Sussan Solymoss**

Canada

**Nikunj Somia**

USA

**Stig Somme**

USA

**Claudia Sommer**

Germany

**Sarah Sonsthagen**

USA

**Jessica Sontrop**

Canada

**Sajid Soofi**

Pakistan

**Nikolai Sopko**

USA

**Andrej Sorgo**

Slovenia

**Peter Sörös**

Canada

**Enea Spada**

Italy

**Kirsten Spann**

Australia

**Michael Spear**

USA

**David Speers**

Australia

**Thomas Spentzas**

USA

**Henry M. Spotnitz**

USA

**Chandrashekhar Sreeramareddy**

Nepal

**Saranya Sridhar**

UK

**Bharath Srinivasan**

USA

**Kamna Srivastava**

India

**Sanjeeva Srivastava**

India

**Vibhuti Srivastava**

USA

**Leanna J. Standish**

USA

**Chalrles Stanley**

USA

**Giorgio Stanta**

Italy

**Jerod Stapleton**

USA

**Renee Stark**

Germany

**Angela Starkweather**

USA

**Martha Starr**

USA

**Lukas Staub**

Switzerland

**Jürgen Stech**

Germany

**Ioannis Stefanidis**

Greece

**Eirikur Steingrimsson**

Iceland

**Elizabeth Stenhouse**

UK

**Peter Stenson**

UK

**Timothy Stephenson**

UK

**Andy Stergachis**

USA

**Bobbie Sterling**

USA

**Lotte Steuten**

The Netherlands

**Diana Stewart**

USA

**Michael Stewart**

USA

**Samuel Alan Stewart**

Canada

**Andrej Steyer**

Slovenia

**Claire Stocker**

UK

**Juan Carlos Stockert**

Spain

**Timothy Stockwell**

USA

**Marcus Stoetzer**

Germany

**Chris Stone**

USA

**William Stone**

USA

**Sabine Stordeur**

Belgium

**Alasdair Strachan**

UK

**Mark Strand**

USA

**Gregory Strayhorn**

USA

**Costin Teodor Streba**

Romania

**Catherine Striley**

USA

**Pablo Strobl-Mazzulla**

Argentina

**Mark Strudwick**

Australia

**Robert Stupar**

USA

**Nancy Sturman**

Australia

**Feng Wen Su**

Taiwan

**Zhenqiang Su**

USA

**Anbukkani Subbian**

India

**Arulselvi Subramanian**

India

**Sujha Subramanian**

USA

**Alan Sugar**

USA

**Minetaka Sugiyama**

Japan

**Mark Sujan**

UK

**Giorgia Sulis**

Italy

**Joanna Sumner**

Australia

**Walton Sumner**

USA

**Samantha Sundercombe**

Australia

**Bruce Sunderland**

Australia

**George Sundin**

USA

**Hoon Sunwoo**

Canada

**Siti Suraiya**

Malaysia

**Johari Surin**

Malaysia

**Leonardo Susta**

Canada

**Keiichiro Susuki**

USA

**Melissa Suter**

USA

**Erling Svensen**

Norway

**Kunchithapadam Swaminathan**

Singapore

**Shivalingappa Swamynathan**

USA

**Bryan Sykes**

USA

**Christopher Ta**

USA

**Loni Tabb**

USA

**Changiz Taghibiglou**

Canada

**Yu Tahara**

Japan

**Leila Taher**

Germany

**Kudakwashe Collin Takarinda**

Zimbabwe

**Tomohiko Taki**

Japan

**Simbarashe Takuva**

South Africa

**Kawsar Talaat**

USA

**Antoine Talarmin**

France

**Jeffery Talbert**

USA

**Jantjie Taljaard**

South Africa

**Audrey Talley Rostov**

USA

**Dinesh Talwar**

UK

**Nicoladie Tam**

USA

**Jyoti Tamang**

India

**Karin Tamar**

Israel

**Ghee Tan**

USA

**Haiping Tan**

Australia

**Jin Ai Mary Anne Tan**

Malaysia

**Tian Chye Tan**

Malaysia

**Ryan Tandjung**

Switzerland

**Chanderdeep Tandon**

India

**Ravi Tandon**

India

**Sheng-Ce Tao**

China

**Mark Tarnopolsky**

Canada

**Marc Tatar**

USA

**Andrey Tatarenkov**

USA

**Ehsan Tavakkoli**

Australia

**Kouhyar Tavakolian**

USA

**Francis Tayie**

USA

**Anthony Taylor**

UK

**Brandie Taylor**

USA

**Molly Taylor**

UK

**Myra Taylor**

South Africa

**Denis Tebit**

USA

**Yonatal Mesfin Tefera**

Ethiopia

**Romina Tejada**

Peru

**David Templeton**

Australia

**Andrew Teodorczuk**

UK

**Henriëtte Ter Waarbeek**

The Netherlands

**Margarita Teran-Garcia**

USA

**Yves Terrat**

Canada

**Christi Terry**

USA

**Gerasimos Terzis**

Greece

**Traci Testerman**

USA

**Ian Joseph Tetlow**

Canada

**Amit Tevar**

USA

**Megan Teychenne**

Australia

**Alana M. Thackray**

UK

**Badri Thapa**

Nepal

**Jagan Mohan Tharakan**

India

**Kassiani Theodoraki**

Greece

**George Therapondos**

USA

**Oliver Michel Theusinger**

Switzerland

**Stephen Thielke**

USA

**Sarat Thikkurissy**

USA

**Aliki Thomas**

Canada

**Rachel Thomson**

Australia

**David Ian Thurnham**

UK

**Thomas Thurnheer**

Switzerland

**Bharat Thyagarajan**

USA

**Thankam Thyvalikakath**

USA

**Luca Tiano**

Italy

**Peter Tilley**

Canada

**Julie K. Tilson**

USA

**Jozsef Timar**

Hungary

**Claudio Tiribelli**

Italy

**Franklin Toapanta**

USA

**Els Tobback**

Belgium

**Michael Todd**

USA

**Ugur Tokay**

Turkey

**Barry Tolchard**

Australia

**David Tollervey**

UK

**Hanna Tolonen**

Finland

**Fernanda Tomazoni**

Brazil

**Laurie Tomlinson**

UK

**Connie Tompkins**

USA

**Jingou Tong**

China

**Qingchun Tong**

USA

**Steven Tong**

Australia

**Yue Tong**

China

**Lil Tonmyr**

Canada

**Maria Regina Torloni**

Brazil

**Belen Torondel**

UK

**Ellen Toth**

Canada

**Michelle Tourigny**

Australia

**Pierre-Louis Toutain**

France

**Elizabeth Tracey**

Australia

**Phong Tran**

USA

**Tinna Traustadottir**

USA

**Jeanette Trauth**

USA

**Jeffrey Travers**

USA

**Jason Treberg**

Canada

**Indi Trehan**

USA

**Julien Tremblay**

Canada

**Mary Trepka**

USA

**Chanwit Tribuddharat**

Thailand

**Jeffrey M. Trimarchi**

USA

**Abhai Tripathi**

USA

**Timir Tripathi**

India

**Emiliano Trucchi**

Austria

**Hedda Tschudi-Madsen**

Norway

**Aster Tsegaye**

Ethiopia

**Fan-Chen Tseng**

Taiwan

**Cleon Tsimbos**

Greece

**Patrick Valere Tsouh Fokou**

Ghana

**Yaping Tu**

USA

**Madalina Tuluc**

USA

**Delphine Tuot**

USA

**Janis Tupesis**

USA

**Renee Turner**

Australia

**Anne Bjørg Tveit**

Norway

**Marc Twagirumukiza**

Belgium

**Nana Twum-Danso**

USA

**Iris Udasin**

USA

**Muhammad Jasim Uddin**

Bangladesh

**Mitsuhiro Ueda**

Japan

**Cesar Ugarte-Gil**

Peru

**Nkolika Uguru**

Nigeria

**Jane Umeano**

Nigeria

**M. Renee Umstattd Meyer**

USA

**Stephan Urban**

Germany

**Tim Usherwood**

Australia

**Bettina Utz**

Belgium

**Francisco Uzal**

USA

**Viveka Vadyvaloo**

USA

**Mandana Vahabi**

Canada

**Annukka Vainio**

Finland

**Edi Vaisbuch**

USA

**Sylvia Valdezate**

Spain

**Raphael Valdivia**

USA

**Veronica Vallejo-Ruiz**

Mexico

**Josien van den Noort**

The Netherlands

**Andre Van der Merwe**

South Africa

**Eveline van der Veer**

The Netherlands

**Wilbert Van Panhuis**

USA

**Moritz van Vuuren**

South Africa

**Joachim Vandekerckhove**

USA

**Timothy VanWagoner**

USA

**Badri Vardarajan**

USA

**Jaira Vasconcellos**

USA

**Andrey Vasin**

Russian Federation

**Senthilvel Vasudevan**

Saudi Arabia

**Ryash Vather**

New Zealand

**Gilberto Vaughan**

USA

**Jefferson Vaughan**

USA

**Gayatri Vedantam**

USA

**Jurgen Veeck**

Germany

**Gary Velan**

Australia

**Norma Velázquez-Guadarrama**

Mexico

**Craig Velozo**

USA

**Bhuvaneswari Velumani**

India

**Shannon Venance**

Canada

**Kavita Venkataraman**

Singapore

**Musturi Venkataramana**

India

**Rengaraj Venkatesh**

India

**Vishwanath Venketaraman**

USA

**Estelle Venter**

South Africa

**Wouter Verbeke**

Belgium

**C. Verdugo**

USA

**Howard Vernon**

Canada

**Beata Vertessy**

Hungary

**Ştefan Vesa**

Romania

**Celine Vetter**

USA

**Nereo Vettoretto**

Italy

**Isabelle Viana**

USA

**Jorge Vidal**

USA

**Michele Vidotto**

Italy

**Cristina Vieira**

Brazil

**Cheryl Vigen**

USA

**Marco Vigilato**

Peru

**Ramachandran Vignesh**

India

**Sanjay Vikrant**

India

**Fidel Vila-Rodriguez**

Canada

**Alan Villavicencio**

USA

**Agnes Vitry**

Australia

**Antonia Vlahou**

Greece

**Čubrić Vlatka**

Croatia

**Beatrix Volc-Platzer**

Austria

**Rüdiger von Eisenhart-Rothe**

Germany

**Ulrich von Pawel-Rammingen**

Sweden

**Frauke von Versen-Höynck**

Germany

**Steve Walczak**

USA

**Linda Waite**

USA

**Lorraine Walker**

USA

**Judy D. Wall**

USA

**Kristin Wall**

USA

**Helle Wallach Kildemoes**

Denmark

**Lance Waller**

USA

**Tony Walls**

New Zealand

**Zach Walsh**

Canada

**Mwanda Walter**

Kenya

**Ryan Walter**

USA

**Katherina Walz**

USA

**Richard Wamai**

USA

**Sidika Wambani**

Kenya

**Ying Wan**

USA

**Chunling Wang**

China

**Debin Wang**

China

**Frederick Wang**

USA

**Jiaqi Wang**

China

**Jing Wang**

USA

**Ju Wang**

USA

**Li Wang**

USA

**San Ming Wang**

USA

**Shuai Wang**

Hong Kong

**Shu-Hua Wang**

USA

**Wenfang Wang**

USA

**Wenyi Wang**

USA

**Xin Wang**

China

**Xin Wang**

USA

**Y. Wang**

USA

**Yan Wang**

USA

**Ying Wang**

USA

**Zhong Wang**

USA

**Phurpa Wangchuk**

Australia

**Jessica Wang-Rodriguez**

USA

**Kenneth Ward**

USA

**Mary Ward**

UK

**Nicole Ward**

USA

**Klaas Wardenaar**

The Netherlands

**Gamal Wareth**

Germany

**Christopher Warlick**

USA

**Steven Warsof**

USA

**Peter Wasswa**

Uganda

**Michael Webb**

UK

**Allison Webel**

USA

**Keith Weeks**

UK

**Christoph Wehmeyer**

Germany

**Adi Yehuda Weintraub**

Israel

**Thomas Weiser**

USA

**Jennifer Weiss**

USA

**Martha Welch**

USA

**Greg Welk**

USA

**Cherie Wells**

Australia

**Michael Wells**

UK

**Xiaozhong Wen**

USA

**Aaron Wendelboe**

USA

**Mark Wener**

USA

**Nomi Werbeloff**

UK

**Andrea Wessell**

USA

**Johanna Westra**

The Netherlands

**Olwyn Westwood**

UK

**Darrell Wheeler**

USA

**Michael Whitby**

Australia

**Laura White**

USA

**Nathan White**

USA

**Robert White**

USA

**Dean Whitehead**

New Zealand

**David Whitford**

Bahrain

**Eric Wickel**

USA

**Silke Wiedmann**

Germany

**Alicja Wiercinska-Drapalo**

Poland

**Lothar Wiese**

Denmark

**Andrew Wilber**

USA

**Luann Wilkerson**

USA

**Katalin Wilkinson**

South Africa

**Thomas Willems**

USA

**Michele Williams**

USA

**Olajide Williams**

USA

**Veronika Williams**

UK

**Kathleen Williamson**

USA

**E. Vance Wilson**

USA

**Jonathan Wilson**

Portugal

**Patricia Wilson**

UK

**Peter Wilson**

UK

**Stuart Wilson**

UK

**Johanna Winberg**

Sweden

**Christopher Winfree**

USA

**Volker Winkler**

Germany

**Bryan Winn**

USA

**Barbara Wirostko**

USA

**Tibor Wittmann**

Hungary

**Kenneth Witwer**

USA

**Viroj Wiwanitkit**

Thailand

**Benchawan Wiwatanapataphee**

Australia

**Kama Wlodzimirow**

The Netherlands

**Robert Wojciechowski**

USA

**Joseph Wolfsdorf**

USA

**Benjamin Wolozin**

USA

**Albert Wong**

Canada

**Cynthia Wong**

USA

**Derrick Wood**

USA

**Nick Woodier**

UK

**Andrew Woolnough**

Australia

**William Worodria**

Uganda

**Devina Worsley**

UK

**Heather Worth**

Australia

**Colin Wright**

UK

**John Wright**

USA

**Kelly Wright**

USA

**Laura Wyatt**

USA

**Alidianne Xavier**

Brazil

**Zheng Xia**

USA

**Xiaoling Xiang**

USA

**Hong Xiao**

USA

**Yonghong Xiao**

China

**Glen Xiong**

USA

**Lin Xu**

China

**Yong Xu**

China

**Zhi-Xiang Xu**

USA

**Han Xue**

China

**Pengcheng Xun**

USA

**Tomio Yabe**

Japan

**Koshika Yadava**

USA

**Mark Yaffe**

Canada

**Hayley Yaglom**

USA

**Wing Cheong Yam**

China

**Masamitau Yamaguchi**

Japan

**Takuji Yamamoto**

Japan

**Yoshio Yamaoka**

Japan

**Zheng Yan**

USA

**Angel Yanagihara**

USA

**Hyun Suk Yang**

South Korea

**Linsheng Yang**

China

**Nan-Ping Yang**

Taiwan

**Tianbing Yang**

USA

**Cedric Yansouni**

Canada

**Beverly Yashar**

USA

**Aymen Yassin**

Egypt

**Zhiwei Ye**

USA

**Kojo Yeboah-Antwi**

USA

**Sailu Yellaboina**

India

**Seonae Yeo**

USA

**Shan Yin**

USA

**Kyong-Ah Yoon**

South Korea

**Ian York**

USA

**Kenichi Yoshida**

Japan

**Kosuke Yoshihara**

Japan

**Jennifer Yost**

Canada

**Y. Nancy You**

USA

**Amanda Young**

UK

**April Young**

USA

**Christopher Young**

Australia

**Jo-Anne Young**

USA

**Jae-Hyuk Yu**

USA

**Jun Yu**

UK

**Tong Yue**

China

**Seok Joong Yun**

South Korea

**Evy Yunihastuti**

Indonesia

**Flora Zagouri**

Greece

**Siti Zain**

Malaysia

**Rubeena Zakar**

Germany

**Zuraini Zakaria**

Malaysia

**Hasniza Zaman Huri**

Malaysia

**Keivan Zandi**

Malaysia

**Gianluigi Zanetti**

Italy

**Hassan Zaraket**

Lebanon

**Amrapali Zaveri**

Germany

**John Zelenski**

Canada

**Boris Zemelman**

USA

**Erliang Zeng**

USA

**Qi Zeng**

USA

**Maria-Christina Zennaro**

France

**Juan Carlos Zenteno**

Mexico

**Daniel Zerbino**

UK

**Farid Zerimech**

France

**Chunbo Zhang**

China

**Chunhua Zhang**

USA

**Dapeng Zhang**

USA

**Guicheng Zhang**

Australia

**Jie Zhang**

USA

**Shuping Zhang**

USA

**Xiang-Dong Zhang**

USA

**Xiaojian Zhang**

China

**Xiaoying Zhang**

China

**Ying Zhen**

USA

**Cheng Zheng**

China

**Cuncong Zhong**

USA

**Li Zhong**

USA

**Qiuyue Zhong**

USA

**Beiyan Zhou**

USA

**Huiyu Zhou**

Ireland

**Xuezhong Zhou**

China

**Yebin Zhou**

USA

**Yi Zhou**

USA

**Qihui Zhu**

USA

**Xiaofei Zhu**

China

**Yeyi Zhu**

USA

**John Ziegler**

Australia

**Robert Zink**

USA

**Melissa Zullo**

USA

**Krystle Zuniga**

USA

**Simbarashe Peter Zvada**

South Africa

**Laurence Zwiebel**

USA

